# LC-MS/MS multiplex analysis of lysosphingolipids in plasma and amniotic fluid: A novel tool for the screening of sphingolipidoses and Niemann-Pick type C disease

**DOI:** 10.1371/journal.pone.0181700

**Published:** 2017-07-27

**Authors:** Magali Pettazzoni, Roseline Froissart, Cécile Pagan, Marie T. Vanier, Séverine Ruet, Philippe Latour, Nathalie Guffon, Alain Fouilhoux, Dominique P. Germain, Thierry Levade, Christine Vianey-Saban, Monique Piraud, David Cheillan

**Affiliations:** 1 Service de Biochimie et Biologie Moléculaire Grand Est, Unité Médicale Pathologies Métaboliques, Erythrocytaires et Dépistage Périnatal, Centre de Biologie et de Pathologie Est, Hospices Civils de Lyon, Bron, France; 2 Unité Mixte de Recherche 5305, Centre National de la Recherche Scientifique (CNRS) Université Claude Bernard Lyon 1, Lyon, France; 3 Unité 820, Institut National de la Santé et de la Recherche Médicale (INSERM), Lyon, France; 4 Laboratoire Gillet-Mérieux, Centre de Biologie et Pathologie Est, Hospices Civils de Lyon, Bron, France; 5 Service de Biochimie et Biologie Moléculaire Grand Est, Unité Médicale Pathologies neurologiques et cardiologiques, Centre de Biologie et de Pathologie Est, Hospices Civils de Lyon, Bron, France; 6 Centre de référence des Maladies Héréditaires du Métabolisme, Hôpital Femme Mère Enfant, Hospices Civils de Lyon, Bron, France; 7 Service de Génétique Médicale et Unité Mixte de Recherche 1179, Institut National de la Santé et de la Recherche Médicale (INSERM), Université de Versailles, Montigny, France; 8 Centre Hospitalo-Universitaire de Toulouse, Institut Fédératif de Biologie, Laboratoire de Biochimie Métabolique, and Unité Mixte de Recherche (UMR) 1037 Institut National de la Santé et de la Recherche Médicale (INSERM), Centre de Recherche en Cancérologie de Toulouse, Toulouse, France; 9 Université de Lyon, Laboratoire CarMeN, Institut National de la Santé et de la Recherche Médicale (INSERM) Unité 1060, Institut National de la Recherche Agronomique (INRA), Unité 1397, Université Claude Bernard Lyon 1, Institut National des Sciences Appliquées (INSA), Lyon, Faculté de médecine Charles Mérieux, Oullins, France; Azienda Ospedaliero-Universitaria Santa Maria della Misericordia, ITALY

## Abstract

**Background:**

The biological diagnosis of sphingolipidoses currently relies on the measurement of specific enzymatic activities and/or genetic studies. Lysosphingolipids have recently emerged as potential biomarkers of sphingolipidoses and Niemann-Pick type C in plasma.

**Methodology:**

We developed a sensitive and specific method enabling the simultaneous quantification of lysosphingolipids by LC-MS/MS: lysoglobotriaosylceramide for Fabry disease, lysohexosylceramide (*i*.*e*. lysoglucosylceramide and/or lysogalactosylceramide) for Gaucher and Krabbe diseases, lysosphingomyelin and its carboxylated analogue lysosphingomyelin-509 for Niemann-Pick type A or B, and C diseases, lysoGM1 ganglioside for GM1gangliosidosis and lysoGM2 ganglioside for GM2 gangliosidosis.

**Findings:**

The diagnostic performances were validated in plasma samples analysing a large series of patients affected with sphingolipidoses and Niemann-Pick type C disease (n = 98), other inborn errors of metabolism (n = 23), and controls (n = 228). The multiplex measurement of lysosphingolipids allowed the screening of Fabry (including female patients and late-onset variants), Gaucher and infantile Krabbe, Niemann-Pick type A/B and C diseases with high sensitivity and specificity. LysoGM1 and LysoGM2 were elevated in most of the patients affected with GM1 and GM2 gangliosidosis respectively.

In amniotic fluid supernatant from pregnancies presenting non-immune *hydrops fetalis* (n = 77, including previously diagnosed Gaucher (n = 5), GM1 gangliosidosis (n = 4) and galactosialidosis (n = 4) fetuses) and from normal pregnancies (n = 15), a specific and dramatic increase of lysohexosylceramide was observed only in the Gaucher amniotic fluid samples.

**Interpretation:**

This multiplex assay which allows the simultaneous measurement of lysosphingolipids in plasma modifies the diagnostic strategy of sphingolipidoses and Niemann-Pick type C. Furthermore, in pregnancies presenting non-immune *hydrops fetalis*, lysohexosylceramide measurement in amniotic fluid offers a rapid screening of fetal Gaucher disease without waiting for glucocerebrosidase activity measurement in cultured amniocytes.

## Introduction

Sphingolipids (SLs) are amphiphilic compounds deriving from a long-chain sphingoid base (mainly sphingosine and sphinganine) that can be *N*-acylated with a variety of fatty acids, to form ceramides. A hydrophilic head group is linked to the hydroxyl group of the sphingoid base via a phosphodiester (phosphosphingolipids) or glycosidic (glycosphingolipids) bond [1]. SLs are essential plasma membrane components of eukaryotic cells and important bioactive molecules [2]. Genetic defects of enzymes or other proteins required for their degradation lead to various lysosomal storage diseases (LSDs) named sphingolipidoses [3]. Clinical manifestations of sphingolipidoses may appear in infancy and childhood, or later during adolescence/adulthood (late-onset forms), but can also present in the antenatal period as non-immune *hydrops fetalis* (NIHF) [4]. A significant secondary storage of SLs also occurs in Niemann-Pick C disease (NPC), characterized by an impaired intracellular trafficking of cholesterol [5]. All these disorders are transmitted on an autosomal recessive mode, except for X-linked Fabry disease (FD). Currently, the diagnosis of sphingolipidoses is based on specific (sometimes complex) enzymatic studies, followed by genetic analyses. For diagnosis purpose, the measurement of SL primarily accumulated in each sphingolipidosis is not relevant in plasma [6], but can be helpful in diseases for which an increase occurs in urine, *i*.*e*., sulfatides in metachromatic leucodystrophy (MLD) [7] or globotriaosylceramide (Gb_3_) in Fabry disease [8].

An increase of the lysosphingolipid (LysoSL, *i*.*e*. SL lacking the *N-*linked fatty acid and thus with a free amino group) of the primary stored compound has been reported in tissues from patients with many sphingolipidoses. Following the initial description of elevated galactosylsphingosine (psychosine) levels in Krabbe disease brain [9,10], LysoSLs have been considered as "toxic metabolites" contributing to the pathophysiology of several sphingolipidoses [11–15], and have been shown to play a variety of pathophysiological roles [16,17]. In recent years, owing to the development of tandem mass spectrometry (MS/MS), their measurement in biological fluids has become possible, and nearly each LysoSL has emerged as a sphingolipidosis biomarker [6,18–22]. Lysosulfatide, not detectable neither in plasma of MLD patients nor in control plasmas, appears as an exception [23]. Finally, a slight elevation of lysosphingomyelin (LysoSM) [24], and a much higher increase of its carboxylated analogue (also called “LysoSM509”) [25] were recently described in plasma of NPC patients.

We have developed a novel LysoSLs multiplex LC-MS/MS assay including for the first time lysoGM1 and lysoGM2 gangliosides (LysoGM1, LysoGM2) for the screening of sphingolipidoses and NPC in a large series of plasma samples. Simultaneous measurement of LysoSLs in plasma (lysoglobotriaosylceramide (LysoGb_3_), lysoglucosylceramide (LysoGlcCer) and lysogalactosylceramide (LysoGalCer) analysed together as lysohexosylceramide (LysoHexCer), lysosphingomyelin (LysoSM), and lysosphingomyelin 509 (LysoSM509)), was recently described in small series of sphingolipidoses [26, 27]. We also performed LysoSLs measurement in amniotic fluid (AF) supernatant in case of NIHF, in order to evaluate the interest of these biomarkers for antenatal screening of sphingolipidoses.

## Materials and methods

### Patients, plasma and AF samples

EDTA Plasma samples were obtained from patients referred for the diagnosis of NPC (n = 8, all NPC1) and sphingolipidoses [n = 90 patients affected with Fabry disease (FD, n = 13 males, n = 29 females), type 1 Gaucher disease (GD, n = 8), Krabbe disease (KD, n = 7 infantile cases), Niemann-Pick type A or B diseases (NPA/B, n = 14), GM1 (n = 6) and GM2 (n = 13) gangliosidoses, and saposin C deficiency (n = 1)], and patients referred for other lysosomal or peroxisomal disorders (n = 23). Control subjects (n = 228) were referred for metabolic investigation but the diagnosis of inborn error of metabolism was discarded. Samples from carriers (parents of GD (n = 2), infantile KD (n = 2) and NPB patients, (n = 2)) were also investigated. In accordance with the French legislation, information was given to patients via the Patient Information Leaflet of our Hospital (Hospices Civils de Lyon, 3, quai des Célestins, 69 229 LYON Cedex 02, France), that was approved by the Hospital Ethics Committee. In this context, verbal non-opposition consent was obtained from all adult subjects and parents of children. Amniotic fluids were obtained after signing a specific consent including diagnostic and research purposes.

For all patients, the diagnosis was established by enzymatic study (sphingolipidoses), or cholestane-3β,5α,6β-triol measurement and filipin staining (NPC), and/or molecular analysis. All samples were obtained before initiation of specific treatment (enzyme replacement therapy (ERT), substrate reduction therapy (SRT) or chaperone therapy), except for the saposin C-deficient patient who was SRT treated. Male FD patients were classified into classical or late-onset variant phenotype according to α-galactosidase A activity, urinary Gb_3_ levels and the *GLA* genotype (see S1 Table). Heterozygous FD females were considered as classical or variant according to the *GLA* mutation. Plasma samples were stored at -20°C for less than 6 months or at -80°C. The stability testing was performed with samples from 10 healthy control subjects for whom a specific informed consent was obtained.

AF samples from 77 pregnancies presenting with NIHF (including AF from fetuses affected with GD (n = 5), GM1 gangliosidosis (n = 4) and galactosialidosis (n = 4) that had previously been diagnosed in our laboratory during a 20 years period by enzymatic studies in cultured amniotic cells) were also investigated. AF samples from 15 other pregnancies, with elevated maternal serum markers of Down syndrome but normal karyotype, were used as controls. AF samples were stored at -20°C until analysis.

### Chemicals

*N-*glycinated-*lyso-*globotriaosylceramide (GlyLysoGb_3_), *lyso-*globotriaosylceramide (LysoGb_3_), D-*erythro*-sphingosyl-phosphorylcholine (lysosphingomyelin d18:1, LysoSM), and *lyso-*monosialoganglioside GM1 (LysoGM1) were from Matreya (State College, PA, USA). D-glucosyl-β1–1′-D*-erythro*-sphingosine (lysoglucosylceramide, LysoGlcCer), D-glucosyl-ß-1,1'-D-*erythro*-sphingosine-d5 (glucosyl(ß)-sphingosine-d5, d5-lysoGlcCer), D-*erythro*-sphingosyl-phosphorylcholine (C17:1 base) (lysosphingomyelin d17:1 LysoSM d17:1) and (2S,3R,4E)-2-amino-1,3-heptadec-4-enediol-1-phosphate (sphingosine-1-phosphate d17:1, S1P d17:1) were from Avanti Polar Lipids, Inc (Alabaster, AL, USA). HPLC grade water was from Ecotainer Aqua B.Braun (Melsungen, Germany). HPLC grade acetonitrile, methanol and formic acid (99%) were from Carlo Erba (Val de Reuil, France). American Chemical Society (ACS) grade ammonium hydroxide [NH_4_OH] (32%) and ACS grade *o-*phosphoric acid [H_3_PO_4_] (85%) were from Merck (Darmstadt, Germany).

### Sample preparation

Stock, and standard solutions were prepared in methanol. Nine points’ standard curves were prepared in methanol. For comparison, similar standard curve were also prepared in plasma matrix. A pool of control plasmas was used for low level quality control (QC). High level QC was prepared by spiking the pool of control plasmas with standards or for LysoSM509, with a pool of plasmas from NPB affected patients.

LysoSLs were extracted by a method adapted from Boutin *et al*. [28]. In brief, 200 μL of plasma or standard samples, or 500 μL of amniotic fluid were mixed with 500 μL of methanol containing 50 nmol/L of each internal standard (IS) (see Table 1 for the corresponding standard and IS) and 500 μL of 2% H_3_PO_4_ in water (v/v). After centrifugation (8 min at 16 000 g), the supernatant was transferred on a mixed-mode cation-exchange solid phase extraction cartridge (Oasis MCX, 30 mg, 60 μm, Waters Corp., Milford, MA, USA), previously washed with 2x600 μL of methanol and 2x600 μL of 2% H_3_PO_4_ in water (v/v). After washing with 2x600 μL of 2% formic acid in water (v/v), LysoSLs were eluted with 2x600 μL of 2% formic acid in methanol (v/v), and 5x600 μL of 2% NH_4_OH in methanol (v/v). After evaporation under nitrogen, samples were reconstituted with 1 mL of 37.5% acetonitrile in water containing 0.2% formic acid and transferred into glass vials for liquid chromatography-tandem mass spectrometry (LC-MS/MS) analysis.

**Table 1:** MS/MS parameters for quantification of lysosphingolipids and their corresponding internal standard (IS).

	Abbreviation IS	MODE	MRM	Declustering Potential (V)	Collision Energy (V)	Retention Time (min)
Lysoglobotriaosylceramide	LysoGb_3_	Positive	786.5 > 282.3	120	44	3.90
	GlyLysoGb_3_ (IS)	Positive	843.5 > 339.3	60	40	3.50
Lysohexosylceramide	LysoGlcCer/LysoGalCer	Positive	462.3 > 282.3	100	30	4.55
	LysoGlcCer d5 (IS)	Positive	467.3 > 287.3	100	30	4.55
Lysosphingomyelin	LysoSM d18:1	Positive	465.3 > 184.0	120	30	4.05
	LysoSM d17:1 (IS)	Positive	451.3 > 184.0	120	30	3.30
Carboxylated analogue of Lysosphingomyelin	LysoSM509 ^a^	Positive	509.3 ^b^ > 184.0	120	30	5.50
	LysoSM d17:1 (IS)	Positive	451.3 > 184.0	120	30	3.30
LysoGM1ganglioside	LysoGM1	Negative	1278.6 > 290.1	-120	-70	3.95
	S1P d17:1 (IS)	Negative	364.2 > 78.9	-60	-70	4.43
LysoGM2 ganglioside	LysoGM2 ^a^	Negative	1116.6 ^c^ > 290.1	-120	-70	4.10
	S1P d17:1 (IS)	Negative	364.2 > 78.9	-60	-70	4.43

Ion source heater temperature = 300°C; source gas 1 = 55 psi; source gas 2 = 50 psi; curtain gas = 20 psi. In positive mode: ion spray voltage = 4500 V; entrance potential (EP) = +10 V; cell exit potential (CXP) = 15 V. In negative mode: ion spray voltage = -4500 V; EP = -10 V; CXP = -15 V.

MRM: multiple reaction monitoring.

^a^ not commercially available.

^b^ from Giese *et al*. [25].

^c^ from Kodama *et al*.[21].

### Liquid chromatography-tandem mass spectrometry

**Fig 1:**
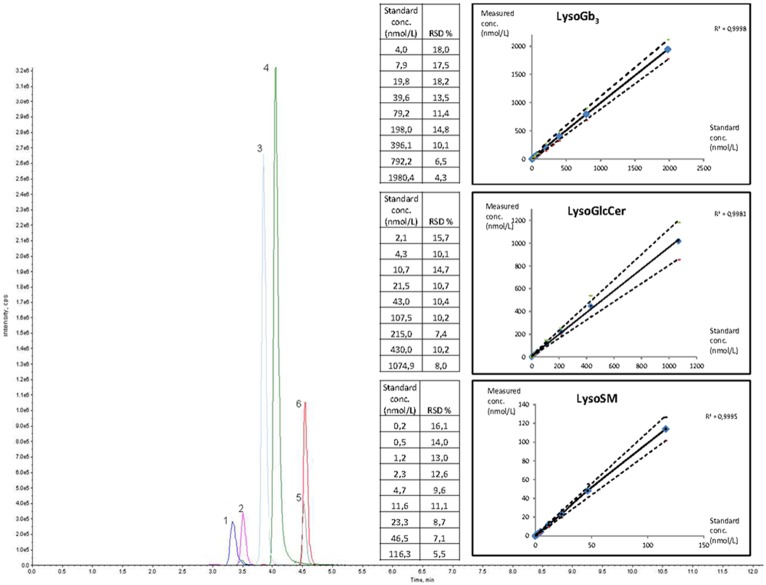
Chromatogram of lysosphingolipids standard solutions and internal standards (IS) in positive mode, standard concentrations, calibration curves, correlation coefficient (R^2^) and relative standard deviation (RSD) of the calibration points. 1: LysoSM d17 (IS); 2: GlyLysoGb_3_ (IS); 3: LysoGb_3_; 4: LysoSM; 5: LysoGlcCer; 6: LysoGlcCer d5 (IS).

**Fig 2:**
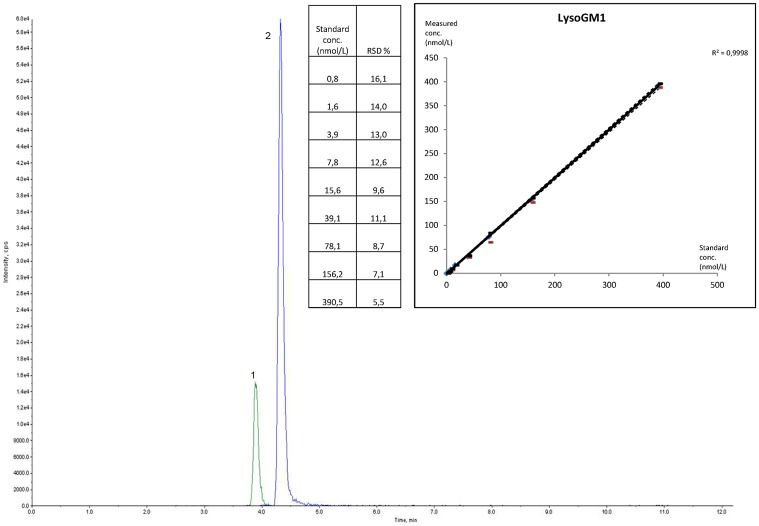
Chromatogram of lysosphingolipids standard solution and internal standard (IS) in negative mode, standard concentrations, calibration curve, correlation coefficient (R^2^) and relative standard deviation (RSD) of the calibration points. 1: LysoGM1; 2: S1P d17 (IS).

LC-MS/MS was performed using two LC pumps (LC20AD) and an auto-sampler (SIL20AC) (Prominence Liquid Chromatograph, Shimadzu, Kyoto, Japan) coupled to an API 4500 QTrap (Applied Biosystems, Concord, Canada) operated in the multiple reaction mode (MRM). MS/MS parameters and retention time (RT) for each molecule are described in Table 1. Positive ionization mode was used for LysoGb_3_, LysoHexCer, LysoSM and LysoSM509, and negative mode for LysoGM1 and LysoGM2 (Figs 1 and 2). Reverse phase LC was performed on a C_8_ column (Uptisphere^®^ 120 Å, 3 μm, 2.1 mm x 50 mm, Interchrom, Interchim, Montluçon, France) maintained at 25°C (phase A: formic acid 0.2% in water, phase B: formic acid 0.2% in acetonitrile). The following gradient was run at a flow rate of 0.4 mL/min. The gradient was initiated at 37.5% of phase A: 0 to 4 min: 37.5 to 50% B, 4 to 8 min: 50% B, 8 to 8.5 min: 50 to 75% B, 8.5 to 9.5 min: 75% B, 9.5 to 10 min: 75 to 37.5% B, 10 to 13 min: 37.5% B. Fifty microliters were injected and the total run time was 13 minutes. Quantification was performed with the Analyst software version 1.6.2, using IS and external calibration curve in 9 points (linear regression, see Figs 1 and 2 for concentrations) except for LysoSM509 and LysoGM2 for which the LysoSM and LysoGM1 calibration curve were used respectively.

### Method validation

Validation was performed according to the ISO EN NF 15189 recommendations [29] as follows. Upper limit of linearity was assessed by spiking the pool of control plasmas with standard solutions. The limit of detection (LoD) was defined as 3 times the standard deviation (SD) of LysoSL/IS ratios of blank samples (n = 10) and the limit of quantification (LoQ) as 10 times the SD of these ratios. Contamination studies were performed for each LysoSL by analysing successively three times the high level QC then three times the low level QC (tenfold lower than the high QC); this sequence was repeated three times and ended by three low level QC. Percentages of the LysoSLs abnormally present in the first low level QC following the last high level QC plasma sample were calculated.

Recovery studies were conducted by spiking 10 plasma samples from healthy controls with 3 levels of standards solutions, the percentage of recovery and the relative standard deviation (RSD) were calculated. Recovery rates within the range 85–115%, and RSD below 20% were considered acceptable. Intra-series (n = 10), and inter-series precision (n = 10) was determined on the two QC levels.

The influence of hemolysis on the lysoSLs results was studied by spiking the pool of control plasmas with hemolysate at various concentrations.

Stability tests and the influence of the anticoagulant agent were evaluated on heparinated plasma, EDTA plasma and serum from 10 healthy controls for LysoGb_3_, LysoHexCer and LysoSM. For stability testing, plasma/serum aliquots were frozen immediately or after 24h and 48h of storage, either at room temperature or at +4°C. For testing total blood stability, blood was centrifuged immediately or stored at room temperature, and centrifuged after 1 day (D1), 2 days (D2), 3 days (D3) or 7 days (D7). Samples were kept frozen at -80°C until used. Biases below 20% were acceptable.

The QCs were used to evaluate the effect of three freeze-thaw cycles. Long term stability was studied on EDTA plasma samples from healthy controls stored up to one year at -20°C or -80°C. The 99^th^ percentile of the control group for LysoGb_3_, LysoHexCer, LysoSM, LysoSM509, and the LoD for LysoGM1 were established as the cut-off (N).

### Statistical analysis

Statistical analysis was performed using JMP^®^ Pro software v10 (SAS Institute Inc., Cary, NC, USA). Non-parametric tests were chosen because of the small group size and the non-normal distribution of variables. Means were compared using the Wilcoxon two-sample test.

## Results

### Method validation

LysoGb_3_, LysoGlcCer, LysoGalCer, LysoSM, and LysoGM1 were easily detectable in standards solution by MS/MS (Table 1, Figs 1 and 2). Isobaric LysoGlcCer and LysoGalCer, displaying similar MS/MS characteristics, could not be separated by our LC procedure (reverse phase LC), and were further analysed as LysoHexCer using LysoGlcCer as standard.

Due to the lack of specific standards, only partial validation was undertaken for lysoSM509 and LysoGM2.

The slopes of the standard curves prepared in methanol or in plasma matrix were not significantly different (<10%) for LysoGb_3_, LysoHexCer and LysoSM (See S1 Fig). The upper limit of linearity, the LoD and LoQ are reported in Table 2. The correlation coefficients (R^2^) calculated from the calibration curves were all above 0.998 (Figs 1 and 2). Intra-series precision was better than 15% and 8% at low and high levels, respectively, except for LysoSM509 (low level: 18%, Table 2). Inter-series precision was below 20% for all compounds at both levels except for LysoGM1 (low level: 23.2%, Table 2). Inter-specimen contamination was ≤ 0.1% for all compounds.

**Table 2:** Validation data of lysosphingolipids measurement in plasma.

	Intra-series Bias (n = 10)				Inter-series Bias (n = 10)				Range of measurement (nmol/L)			Inter-specimen contamination
	Low QC		High QC		Low QC		High QC					
	conc. (nmol/L)	RSD	conc. (nmol/L)	RSD	conc. (nmol/L)	RSD	conc. (nmol/L)	RSD	LoD	LoQ	Limit of Linearity	
LysoGb_3_	4.3	13.8%	60.1	5.5%	4.3	19.0%	60.1	8.9%	0.23	0.40	3960	0.1%
LysoHexCer	0.7	12.2%	38.4	5.4%	0.7	15.7%	38.4	8.3%	0.05	0.16	2150	< 0.1%
LysoSM	1.4	5.1%	6.4	7.1%	1.4	18.7%	6.4	20.0%	< 0.01	0.01	233	< 0.1%
LysoSM509	1.2	18.0%	67.0	6.3%	1.2	19.4%	67.0	14.9%	ND	ND	ND	< 0.1%
LysoGM1	< 0.2	/	15.5	7.8%	< 0.2	/	15.5	23.2%	0.07	0.23	390	< 0.1%

For details, see text. conc.: concentration; LoD: limit of detection; LoQ: limit of quantification; ND: not determined; QC quality control; RSD: relative standard deviation.

### Robustness of the method and impact of preanalytical conditions

Comparing plasma to serum, no bias was observed, except for LysoHexCer (normal values 58% higher in serum, see S3 Table). All LysoSLs tested were stable for up to 48h in plasma or serum, at room temperature or at +4°C (see S4 Table). They were stable up to 24h in total blood on lithium heparin, EDTA, and dry tube (see S4 Table).

At -20°C, all LysoSLs were stable for a period up to 8 months except LysoSM, which clearly increased after 6 months of storage (S2 Fig). When stored at -80°C, all LysoSLs were stable for at least one year. LysoSLs were stable in plasma samples even after three freeze-thaw cycles. No impact of hemolysis was found until 7.9 g/L of hemoglobin (see S5 Table).

### Matrix effects

A solid phase extraction (Oasis MCX cartridge, 30 mg, 60 μm) and an LC step prior to MS/MS analysis were used to reduce potential interference from the matrix, eliminating neutral and acidic compounds and salts before entering the ionization source. Moreover, specific IS (stable isotope-labelled when available, or structurally similar to the LysoSL, see Table 1) were used to correct the potential ion suppression effect. Matrix effect was studied based on recovery studies. The RSD recoveries were validated for LysoGb_3_, LysoGlcCer, and LysoSM but not for LysoGM1 (S2 Table).

### Application to plasma samples

**Fig 3:**
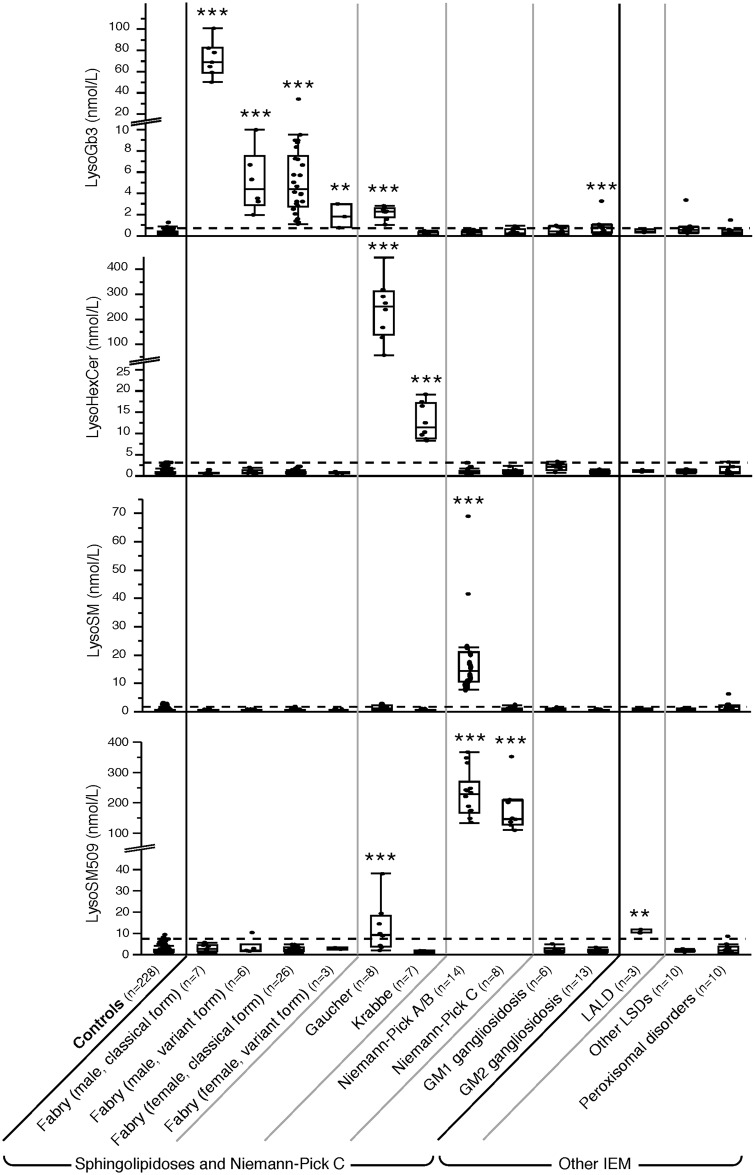
Lysosphingolipids concentrations (nmol/L) in plasma from controls, sphingolipidoses, Niemann-Pick type C disease and other inborn error of metabolism. Statistical significance is indicated as follows: * *p* < 0.05; ** *p* < 0.01; *** *p* < 0.001. LALD: Lysosomal Acid Lipase Deficiency; LSDs: lysosomal storage diseases.

The results of multiplex measurement of LysoGb_3_, LysoHexCer, LysoSM, LysoSM509, LysoGM1, and LysoGM2 in plasma of patients and controls are presented in Table 3 and Fig 3. For details, see S1 Table.

**Table 3:** Lysosphingolipids datas in plasma (nmol/L) from controls, sphingolipidoses, Niemann-Pick C disease and other inborn errors of metabolism.

			LysoGb_3_			LysoHexCer			LysoSM			LysoSM509			LysoGM1 ^a^			LysoGM2 ^a^		
Cut-off (N)			< 0.6			< 3.3			< 1.9			< 7.0			LoD (0.07)			-		
	n	age range (years)	mean	sd	range	mean	sd	range	mean	sd	range	mean	sd	range	mean	sd	range	mean	sd	range
**CONTROLS**	228	0.1–82	0.2	0.2	0.0–1.1	0.8	0.6	0.1–3.5	0.4	0.4	0.1–2.6	1.6	1.5	0.0–9.1	< 0.07			ND		
**SPHINGOLIPIDOSES / NIEMANN-PICK C DISEASE**	98																			
**Fabry disease**	42																			
*- Male*, *classical form*	*7*	0.3–48	**70.7** ***	**17.5**	**47.8–100.8**	0.7	0.4	0.3–1.5	0.3	0.1	0.2–0.4	2.6	1.8	0.2–5.3	< 0.07			ND		
*- Male*, *variant form*	*6*	25–60	**4.8** ***	**2.8**	**1.8–9.4**	0.9	0.6	0.4–2.1	0.3	0.1	0.2–0.4	2.9	3.5	1.1–10.1	< 0.07			ND		
*- Female*, *classical form*	*26*	18–68	**5.6** ***	**6.1**	**1.0–31.2**	0.8	0.6	0.3–2.3	0.3	0.2	0.2–1.3	2.0	1.1	0.6–4.3	< 0.07			ND		
*- Female*, *variant form*	*3*	33–60	**1.7** **	**1.1**	**0.6–2.8**	0.7	0.3	0.4–1.0	0.4	0.1	0.4–0.5	2.3	0.4	2–2.8.0	< 0.07			ND		
**Gaucher disease**	8	1–50	2.0***	0.6	0.9–2.6	224.0***	**119**	**45.7–427**	1.3***	0.7	0.3–2.3	12.4 ***	12.2	1.5–38.7	< 0.07			ND		
**Krabbe disease**	7	0.4–1	0.2	0.1	0.0–0.4	**13.6** ***	**4.7**	**9.3–21.6**	0.3	0.2	0.1–0.5	0.8	0.5	0.1–1.4	< 0.07			ND		
**Niemann-Pick A/B disease**	14	0.7–73	0.2	0.1	0.0–0.5	1.0	0.9	0.4–3.3	**19.9** ***	**16.7**	**8–69.6**	**221.6** ***	**78.1**	**126.8–363.8**	< 0.07			ND		
**Niemann-Pick C disease**	8	0.2–37	0.3	0.3	0.0–0.8	1.0	0.7	0.2–2.4	0.9	0.6	0.3–2.1	**172.1** ***	**79.7**	**101.5–348.6**	< 0.07			ND		
**GM1 gangliosidosis**	6	0.1–26	0.4	0.3	0.1–0.8	2.2	1.0	0.7–3.7	0.6	0.3	0.4–1.2	1.9	1.5	0.3–4.7	**14.3**	**17.8**	**0.0–39.9**	ND		
**GM2 gangliosidosis**	13	0.1–44	0.7 ***	0.8	0.1–3.0	0.8	0.4	0.1–1.6	0.3	0.1	0.1–0.6	1.3	0.8	0.0–3.0	< 0.07			**14.0**	**32.1**	**0.0–118.0**
*Tay-Sachs*	*4*	0.6–44	0.4	0.3	0.2–0.8	1.2	0.4	0.8–1.6	0.4	0.1	0.3–0.6	1.2	0.5	0.6–1.8	< 0.07			**3.9**	**7.1**	**0.0–14.5**
*Sandhoff*	*9*	0.1–40	0.9	0.9	0.1–3.0	0.7	0.4	0.1–1.2	0.3	0.1	0.1–0.4	1.3	0.9	0.0–3.0	< 0.07			**18.6**	**38.0**	**0.0–118.0**
**OTHER INBORN ERRORS OF METABOLISM**	23																			
**LALD**	3	0.2–19	0.3	0.1	0.2–0.5	1.1	0.3	0.9–1.4	0.5	0.3	0.4–0.8	10.6 **	0.9	9.9–11.6	< 0.07			ND		
**Other LSDs**	10	0.1–69	0.7	0.9	0.2–3.1	1.0	0.4	0.5–1.7	0.6	0.2	0.4–0.8	1.4	0.5	0.7–2.3	< 0.07			ND		
**Peroxisomal disorders**	10	0.1–87	0.3	0.4	0–1.3.0	1.3	1.0	0.4–3.5	1.4	1.9	0.2–6.5	2.3	2.5	0.1–8.3	< 0.07			ND		

Other LSDs: Metachromatic leukodystrophy (n = 2), Pompe disease (n = 5), Mucopolysaccharidosis type I (n = 1), Mucopolysaccharidosis type II (n = 1), Mucolipidosis type III (n = 1); Peroxisomal disorders: X-linked adrenoleukodystrophy (n = 3), Adrenomyeloneuropathy (n = 3), Peroxisomal biogenesis defects (n = 4).

Statistical significance is indicated as follows:

*:*p* < 0.05;

**:*p* < 0.01;

***:*p* < 0.001.

The cut-off corresponds to the 99^th^ percentile of controls for LysoGb_3_, LysoHexCer, LysoSM and LysoSM509. In bold: specific increase. LALD: Lysosomal Acid Lipase Deficiency; LoD: limit of detection. LSDs: lysosomal storage diseases. n: number of patients. ND: not detected. sd: standard deviation.

^a^ indicative estimation (poor validation results, see S2 Table).

In the control population, LysoGb_3_, LysoHexCer, LysoSM and LysoSM509 concentrations were very low, but validly measurable while LysoGM1 and LysoGM2 were undetectable. No variation according to sex was found. A slight positive correlation according to age was found for LysoGb_3_ and LysoSM (< 10 years and > 10 years, *p* < 0.01 and *p* < 0.05 respectively), but these differences were not relevant when taking the 99^th^ percentile as cut-off (S6 and S7 Tables). Specific LysoSLs showed a marked elevation in the corresponding sphingolipidosis as well as in NPC (Table 3, Fig 3).

LysoGb_3_ was elevated in all patients with FD. The increase was huge in males with classical form (mean 70.7 nmol/L, *p* < 0.001), milder in females with classical form (5.6 nmol/L, *p* < 0.001) and males with variant form (4.8 nmol/L, *p* < 0.001, *GLA* mutations: p.Phe113Leu, p.Ile198Thr, p.Asn215Ser, p.Arg301Gln, and p.Arg363His), and weak but constant in three females with variant form (mean 1.7 nmol/L, *p* < 0.01, *GLA* mutations: p.Phe113Leu, p.Asn215Ser). LysoGb_3_ was also slightly elevated in all GD samples (mean 2.0 nmol/L, *p* < 0.001), and in 8/13 GM2 gangliosidoses samples (mean 0.7 nmol/L, *p* < 0.001) (for detail see S1 Table).

LysoHexCer was highly increased in all GD patients (mean: 224.0 nmol/L, N < 3.3 nmol/L, *p* < 0.001). In addition, it was elevated in one SRT-treated saposin C-deficient patient (75.8 nmol/L). LysoHexCer was moderately increased in all infantile KD patients (mean: 13.6 nmol/L, *p* < 0.001). An additional peak was systematically observed on the extracted ionic current chromatogram of LysoHexCer with slightly shorter RT than LysoHexCer (see S3 Fig). This peak was absent in GD, in controls, and in patients affected with other lysosomal or peroxisomal disorders.

LysoSM was clearly increased in patients with NPA/B (mean: 19.9 nmol/L, N < 1.9 nmol/L, *p* < 0.001), and not in those with NPC but one (2.1 nmol/L, N < 1.9 nmol/L). A slight elevation could also occur in GD (Table 3, Fig 3).

LysoSM509 was dramatically increased in NPA/B (mean: 221.6 nmol/L, N < 7.0 nmol/L, *p <* 0.001) and NPC (mean: 172.1 nmol/L, *p* < 0.001), and moderately elevated in GD (mean 12.4 nmol/L, *p <* 0.001) and in lysosomal acid lipase deficiency (LALD, mean 10.6 nmol/L, *p <* 0.01).

The sensitivity was 100% for LysoGb_3_ in FD, LysoHexCer in GD and KD, LysoSM in NPA/B, and LysoSM509 in NPA/B and NPC. Non-specific and very moderate elevations were observed in other sphingolipidoses, and were easy to interpret in the context of simultaneous LysoSLs measurement.

LysoGM1 and LysoGM2 were not detectable in controls and patients affected with disorders other than GM1 and GM2 gangliosidoses. LysoGM1 was detected in 5 infantile GM1 gangliosidoses but not in one adult case (Table 3). LysoGM2 was detected in 10 patients with GM2 gangliosidosis (Table 3), but not in one adult Tay-Sachs, 1 adult Sandhoff and 1 infantile Sandhoff patients.

LysoSLs in carriers of GD, infantile KD and NPB were under the cut-off.

### Application to AF samples

**Fig 4:**
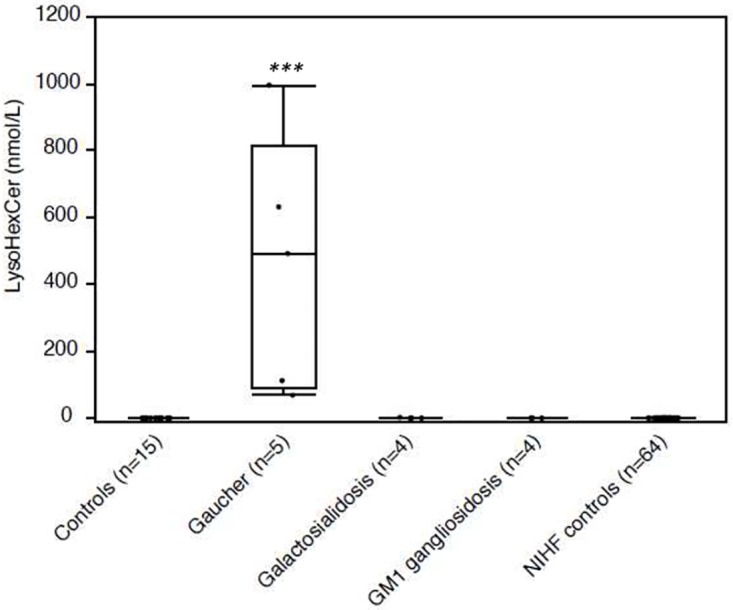
Lysohexosylceramide (LysoHexCer) data in amniotic fluid (AF) supernatant from non-immune hydrops fetalis (NIHF) pregnancies including Gaucher disease, and controls. *** *p* < 0.001.

A major increase of LysoHexCer was observed in the 5 AF supernatants from pregnancies carrying GD affected fetuses (mean 460.1 nmol/L, range 68.4–996.0 nmol/L, *p* < 0.001 Fig 4), but not in the other AF samples (all < 2.0 nmol/L, Table 4). No LysoGM1 was detectable in any of the AF supernatants, including those from fetuses affected with GM1 gangliosidosis (n = 4) or galactosialidosis (n = 4).

**Table 4:** Lysosphingolipids concentrations (nmol/L) in amniotic fluid (AF) supernatant from non-immune hydrops fetalis (NIHF) pregnancies (including Gaucher disease), and controls.

	n	Weeks of gestation (range)		LysoGb_3_	LysoHexCer	LysoSM	LysoSM 509	LysoGM1	LysoGM2
**Control AF**	15	12–31	mean	< 0.4	< 0.7	< 0.1	< 0.9	< 0.07	ND
**Gaucher Disease**	5	20–27	mean	6.8 ***	460.1***	0.1	0.8 ***	< 0.07	ND
			range	0.7–17.2	**68.4–996.0**	< 0.2	0.1–1.9	< 0.07	ND
**GM1 gangliosidosis and galactosialidosis**	8	14–27	mean	< 0.4	< 2.0	< 0.1	< 0.2	< 0.07	ND
**Other causes of NIHF**	64	13–35	mean	< 0.4	< 0.7	< 0.1	< 0.9	< 0.07	ND

Statistical significance is indicated as follows:

*** *p* < 0.001.

n: number of cases. ND. Not detected. In bold: specific increase.

## Discussion

Many reports concerning the measurement in plasma, or dried blood spots, of a single LysoSL have been published [6,18–22,24,25,28,30]. Recently, a multiplex assay of LysoGb_3_, LysoHexCer, LysoSM and LysoSM509 has been described, using one single IS (glucosylsphingosine from plant sources) in a small series of patients (n = 38) [26]. Very recently, Mirzaian *et al*. developed a similar study using ^13^C-labelled IS for LysoGb_3_ and LysoHexCer and C17-LysoSM for LysoSM, in series of FD and GD patients.

In our study, we have extended the panel of LysoSLs to LysoGM1 and LysoGM2, analysing a larger series of sphingolipidoses (n = 90) and NPC (*NPC1*, n = 8), using one specific IS for each LysoSL (Table 1). We also measured LysoSLs in other lysosomal and peroxisomal diseases (see Table 1) to evaluate the specificity of these biomarkers. Applied to AF supernatant, we evaluated the interest of this assay for the screening of sphingolipidoses in a context of NIHF.

In plasma, this rapid method can be used as a more powerful and cost-effective tool for the first-line screening of sphingolipidoses and NPC than other sometimes complex and time-consuming laboratory tests (e.g. galactocerebrosidase and sphingomyelinase activity measurements or filipin staining).

Quantification of LysoGb_3_, LysoHexCer, and LysoSM, is obtained with a good precision and accuracy, and a wide range of physiological and pathological values.

For LysoSM measurement, samples must be stored less than 6 months at -20°C, otherwise at -80°C (see S2 Fig, [24]).

As the standard compound of lysoSM509 is not available, quantification was performed using LysoSM standard curve [25]. As the instrument responses are different between analytes and calibrators, the assay accuracies cannot be evaluated, but only estimated.

In spite of acceptable intra-series precision results for LysoGM1, recovery results were poor (see S2 Table) and quantitative results are thus rough estimations (Table 3). Measurement of LysoGM2 was also attempted with the same limitations.

In FD, our results are in good accordance with previous studies [31]. LysoGb_3_ is elevated in all patients: highly in classical males, and moderately in male variants, classical females or female variants but always above the cut-off value. LysoGb_3_ seems to be more helpful for FD screening than urinary Gb_3_, which is elevated in only 82% of classical females and not in female variants [32]. In FD females, correlation of X inactivation pattern with α-galactosidase A activity has been documented [33].Correlation of α-galactosidase A activity with plasma LysoGb_3_ (and urinary Gb_3_) is not yet established in females, but can be postulated. Should this hypothesis be confirmed, the screening of classic FD in females by measuring plasma LysoGb_3_ might prove the most reliable test.

In GD, LysoGb_3_ is slightly elevated (our study, [26,27,34]) secondary to the activation of acid ceramidase in lysosome [34], overlapping with FD females and male FD variants, but the simultaneous measurement of LysoSLs allows to easily identify GD patients.

LysoGalCer (from Sigma-Aldrich) and LysoGlcCer standard have exactly the same MS/MS characteristics with a high sensitivity but the C8 HPLC column does not allow their separation. Thus, we chose LysoGlcCer standard for quantification, on the basis that Gaucher disease (GD) is much more frequent than Krabbe disease (KD), and often requires follow-up for treatment. Separation is possible for research experiments [19,20,24,26,34]. In case LysoHexCer is found increased in plasma at the first screening step, the diagnosis is always confirmed by measuring appropriate enzymatic activities in leucocytes.

Our results confirm that GD patients display a high increase of LysoHexCer (corresponding to LysoGlcCer)[30]. Plasma LysoHexCer is reported to be correlated with chitotriosidase activity [30,35], but is more reliable as 6–8% of patients present chitotriosidase deficiency (due to a homozygous 24-bp duplication in the *CHIT1* gene) and about 35% are heterozygous for this duplication [36], hampering the interpretation of chitotriosidase levels. Moreover, chitotriosidase is moderately elevated in other lysosomal storage diseases including NPA/B and NPC diseases [37], GM1 gangliosidosis [38], and some FD patients [39], while LysoHexCer is normal; our multiplex method allows the discriminant screening of all these disorders.

LysoHexCer was also found elevated in saposin C deficiency (our case, [30]) and both LysoHexCer and LysoGb_3_ are elevated in prosaposin deficiency [40]. Consequently, the measurement of these plasma biomarkers constitutes today the test of choice before confirmation of the diagnosis by *PSAP* gene analysis. LysoHexCer has not been investigated in saposin A deficiency, nor LysoGM2 in GM2 activator protein deficiency. Measurement of LysoGb_3_ and LysoHexCer can be used for the follow-up of FD and GD therapy respectively [18,30,41–43], and several studies have demonstrated the relationship between LysoSL levels, severity, and age of onset in FD [6,44] or GD [30,35].

As previously reported in dried blood spots [19,20], our study confirms that LysoHexCer is moderately but clearly increased in plasma of infantile KD. Isobar LysoGlcCer and LysoGalCer were not chromatographically separated, but their distinction does not appear mandatory for the differential diagnosis of KD and GD in symptomatic patients considering that: i) clinical presentations are different; ii) higher levels of LysoHexCer are usually present in GD compared to KD (*p* < 0.001); iii) an additional peak (unknown structure, isobar compound of LysoGalCer? see S3 Fig) was observed in all KD patients but not in GD, iv) subsequent determination of glucocerebrosidase/galactocerebrosidase activities would allow to establish the diagnosis.

We observed a combined increase of LysoSM and LysoSM509 in plasma of NPA/B patients, whereas high LysoSM509 without increase of LysoSM (0.2–1.1-fold) was found in NPC. Earlier studies had shown a significant increase of LysoSM509 in plasma of patients with NPC or NPA/B, and of LysoSM in dried blood spots or plasma of NPA/B patients, with only a small or no increase of LysoSM in NPC [22,24–26,45]. The combined study of both biomarkers thus allows discrimination between these two disorders, as recently discussed [26,46]. However, it seems that LysoSM in plasma can be nearly normal in some mildly affected NPB patients [47]. A slight increase of LysoSM509 (5/8 patients) and of LysoSM (2/8 patients) was observed in GD cases (Table 3), in accordance with recent findings [26,27,34]. In a context of isolated (hepato)splenomegaly, especially in neonates, the co-measurement of LysoSLs in plasma provides the possibility to screen for GD (LysoHexCer), NPA/B (LysoSM,LysoSM509) and NPC (LysoSM509) diseases in a single run, using a small blood volume.

In our series, LysoGM2 was increased in plasma from 10/13 GM2 patients; this elevation was previously observed by Kodama *et al*. with an HPLC method [21]. LysoGM1 was increased in 5/6 GM1 gangliosidosis patients. LysoGM1/GM2 false negative patients (4/19 cases) were mostly late-onset forms (3/4 cases). LysoGM1/GM2 were not detectable, neither in controls, nor in the other disorders. Increasing the sensitivity of the equipment could improve the sensitivity of detection, and using an adequate isotope labelled IS could allow accurate quantification. However, the analysis of LysoGM1/GM2 associated with other LysoSLs in plasma can be useful and a time-saving way to investigate simultaneously many LSDs.

The sensitivity of each marker was 100% for the corresponding disorders except for GM1 and GM2 gangliosidoses (83% and 77% respectively). The risk of misdiagnosis possibly due to MS/MS instrument fluctuation, and to the fouling of the apparatus, is controlled for LysoGb3, LysoHexCer, LysoSM and LysoSM509 in each series: i) by checking LysoSL peaks are higher than the LOQ for control samples (the peak of LysoGM1 and LysoGM2 is absent in control samples, thus, the risk of misdiagnosis only affects the GM1/2 gangliosidoses), ii) by daily cleaning of the apparatus, iii) by checking the stability of the IS area over time, iv) by the use of QCs.

The specificity of LysoSLs was evaluated by testing plasma from GD, infantile KD and NPB carriers, and patients affected with other metabolic diseases (Table 3, Fig 3). For carriers, LysoSLs levels were all within control ranges; in peroxisomal disorders, LysoSLs were found very slightly elevated; in some LSDs, a slight and non-specific increase was observed: LysoGb_3_ in GM2 gangliosidosis patients, and interestingly LysoSM509 in LALD patients. Possibly, the metabolic block affects the global degradation pathway of sphingolipids. Several other factors could be responsible for LysoSLs unspecific increases: cationic amphiphilic drugs like amiodarone can induce lipidosis [31]; LysoSM can also be elevated in the plasma of patients with metabolic syndrome [48], and a lowering effect of oral contraceptives on LysoHexCer was recently described [27]. These elevations however are moderate when compared to those observed in sphingolipidoses and NPC. Only a slight increase of LysoGb_3_ must be interpreted with caution.

No late-onset KD could be investigated in our study. It has been earlier demonstrated that LysoGalCer in dried blood spots was lower in late-onset KD than in neonatal KD [20]. Thus, a normal LysoHexCer in plasma might be expected in late-onset KD, and possibly in saposin A deficiency [49].

Some sphingolipidoses (GD, GM1 gangliosidosis, and more rarely NPA, and Farber disease, [50–52]) and NPC [53] can present with antenatal signs, mainly NIHF. Today, the screening of LSDs in case of NIHF is usually performed by analysing oligosaccharides, free sialic acid, and glycosaminoglycans in AF supernatant, and by measuring enzymatic activities or performing filipin staining in cultured amniotic cells [4,54,55]. We applied our LysoSLs measurement assay to AF samples from pregnancies carrying LSD affected fetuses previously diagnosed in our laboratory. Unlike LysoGM1, detected neither in controls nor in GM1 gangliosidosis or galactosialidosis AF samples, LysoHexCer showed a dramatic and specific increase in AF from fetuses affected with GD (Table 4, Fig 4). Thus, measuring LysoHexCer in AF represents a new powerful analysis for fetal GD screening, which needs to be confirmed by the glucocerebrosidase activity measurement in cultured amniotic cells.

## Conclusion

The simultaneous analysis of LysoSLs allows the efficient screening of sphingolipidoses and NPC in plasma. Whereas the diagnosis must always be confirmed by appropriate enzymatic/genetic analysis, this approach greatly modifies the diagnostic algorithm for these disorders. When applied to AF supernatant, LysoHexCer quantification can be used as the first tier method for the screening of fetal GD in pregnancies presenting with NIHF, giving more rapid results than the glucocerebrosidase activity measurement in cultured amniocytes. However, the use of this technique for the screening of fetuses affected with other sphingolipidoses or NPC remains to be evaluated.

## Supporting information

S1 Fig Lysosphingolipids calibration curves in methanol and in plasma matrix. S1-1 Lysoglobotriaosylceramide (LysoGb_3_). S1-2 Lysoglucosylceramide (LysoGlcCer). S1-3 Lysosphingomyelin (LysoSM). (PDF)

S2 Fig Lysosphingomyelin concentrations in control plasmas analysed after a variable time of storage. (PDF)

S3 Fig Extracted ionic current chromatograms of lysohexosylceramide in plasma (positive mode). S3-1 Control, S3-2 Gaucher disease, S3-3 Infantile Krabbe disease. The arrow indicates a second peak, observed only in infantile Krabbe Disease.
(PDF)

S1 Table Data for patient affected with sphingolipidoses, Niemann-Pick C and other inborn error of metabolism (age, sex, and lysosphingolipids results). For Fabry disease, *GLA* gene results are presented.
(DOCX)

S2 Table Recoveries (%) and relative standard deviation (RSD. %) after spiking of 10 control plasmas with standard solutions at low, medium and high levels. Recovery rates within the range of 85–115%. and RSD below 20% were considered acceptable.
(DOCX)

S3 Table Influence of the anticoagulant agent on lysosphingolipids results (mean of 10 healthy controls, nmol/L). Biases below 20% were considered acceptable.
(DOCX)

S4 Table Lysosphingolipids data in aliquots frozen immediately or after different time of storage, at room temperature or at + 4°C (mean of 10 healthy controls, nmol/L). Biases below 20% were considered acceptable.
(DOCX)

S5 Table Impact of hemolysis on lysosphingolipids concentrations in plasma (nmol/L). (DOCX)

S6 Table Influence of sex on lysosphingolipids results (nmol/L). NS: non-significant.
(DOCX)

S7 Table Influence of age on lysosphingolipids results (nmol/L). NS: non-significant. y: year.
(DOCX)
