# Supplementary figures and images for: LC-MS/MS multiplex analysis of lysosphingolipids in plasma and amniotic fluid: A novel tool for the screening of sphingolipidoses and Niemann-Pick type C disease
Source: PLoS One. 2017 Jul 27;12(7):e0181700. doi: 10.1371/journal.pone.0181700 (PMC5531455; doi:10.1371/journal.pone.0181700)

S1\_Fig

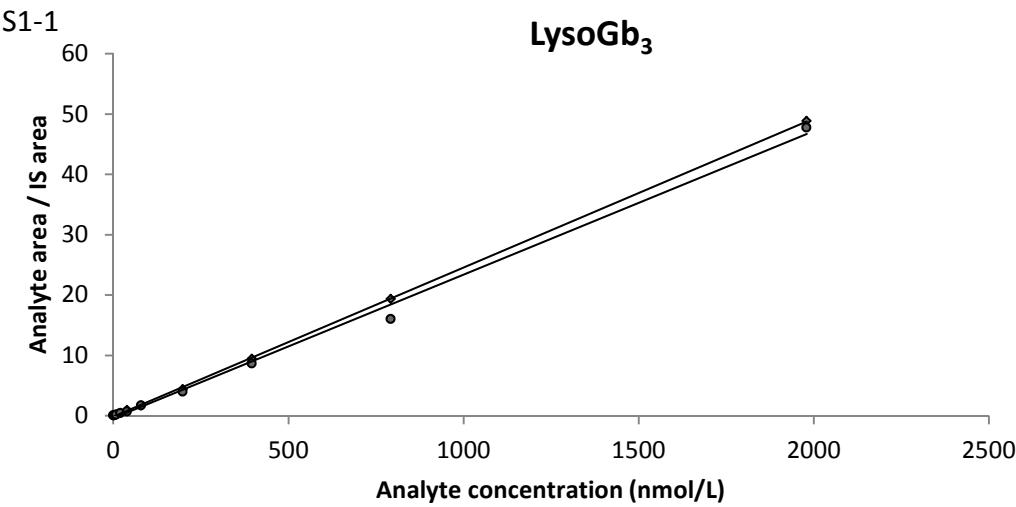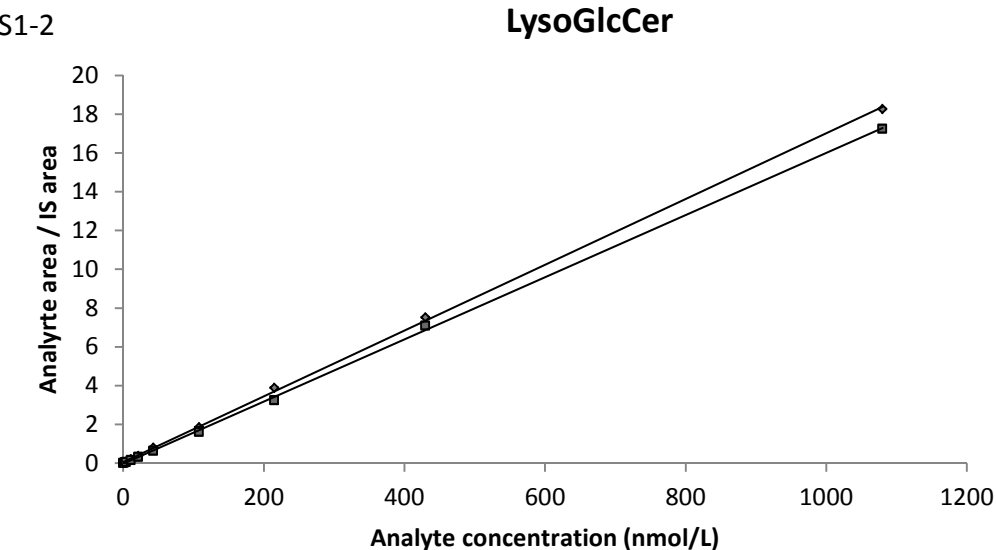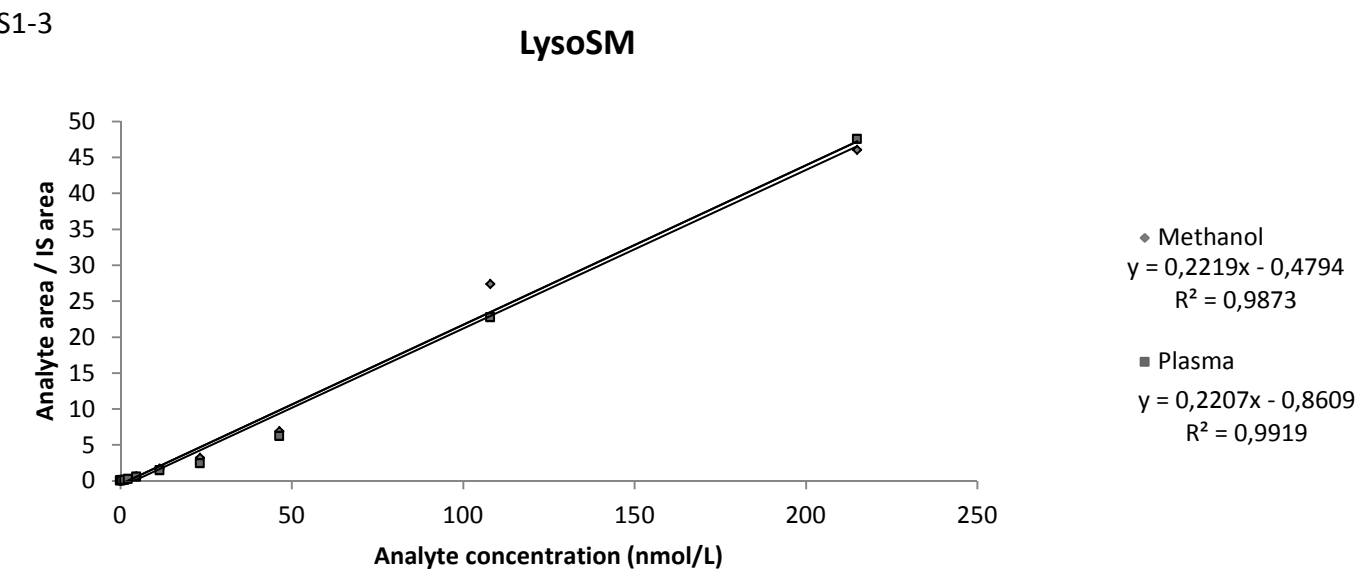

Supplement: S1 Fig — (PDF) [file pone.0181700.s001.pdf]

S2\_Fig

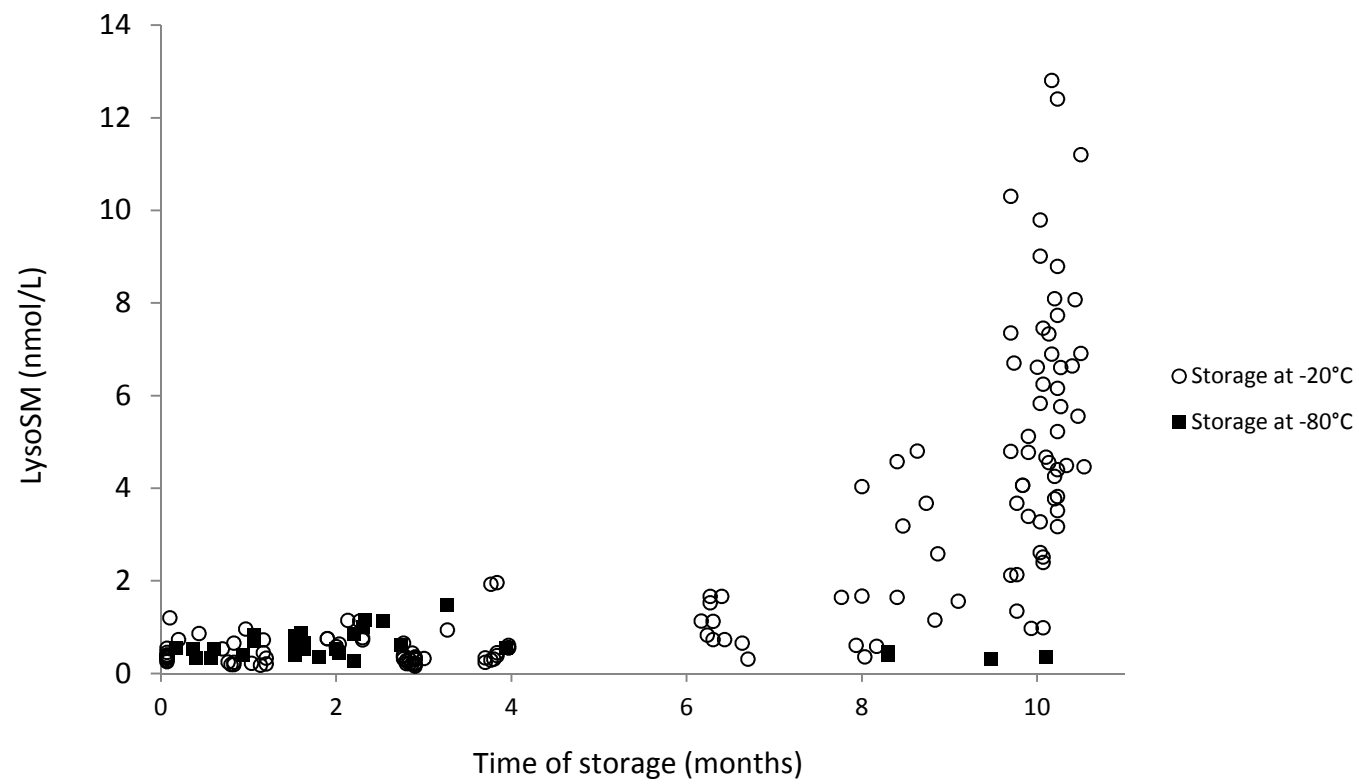

Supplement: S2 Fig — (PDF) [file pone.0181700.s002.pdf]

S3\_Fig

S3-1

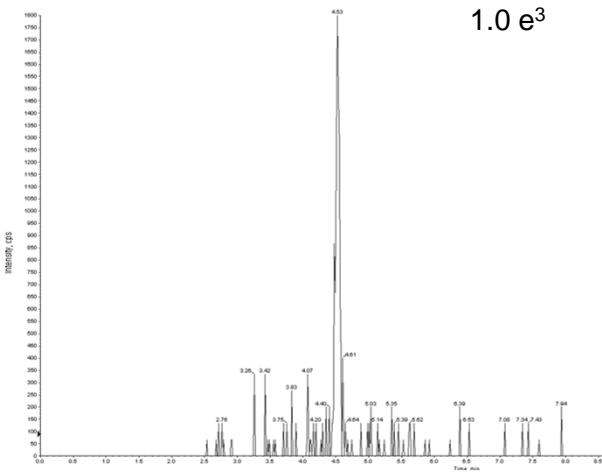

S3-2

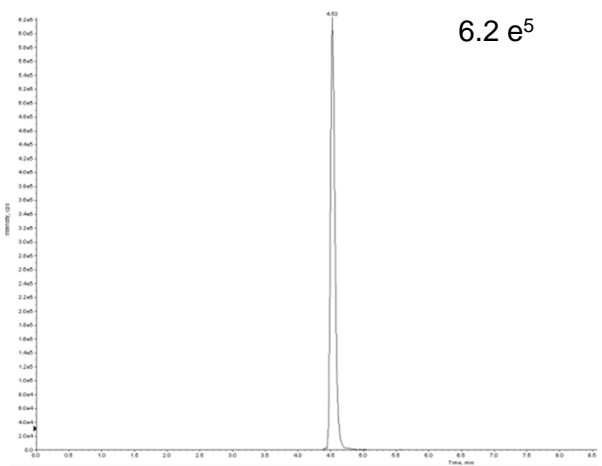

S3-3

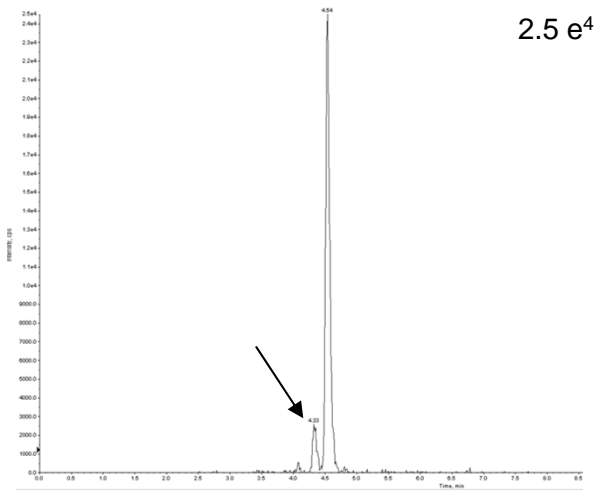

Supplement: S3 Fig — S3-1 Control, S3-2 Gaucher disease, S3-3 Infantile Krabbe disease. The arrow indicates a second peak, observed only in infantile Krabbe Disease. (PDF) [file pone.0181700.s003.pdf]
