# Supplementary material for: LC-MS/MS multiplex analysis of lysosphingolipids in plasma and amniotic fluid: A novel tool for the screening of sphingolipidoses and Niemann-Pick type C disease
Source: PLoS One. 2017 Jul 27;12(7):e0181700. doi: 10.1371/journal.pone.0181700 (PMC5531455; doi:10.1371/journal.pone.0181700)
Supplement: S1 Table — For Fabry disease, GLA gene results are presented. (DOCX) [file pone.0181700.s004.docx]

| **SPHINGOLIPIDOSES AND NIEMANN-PICK C DISEASE** | | | | | |  |  |  |  |  |  |
| --- | --- | --- | --- | --- | --- | --- | --- | --- | --- | --- | --- |
| **Fabry disease** | |  |  |  |  |  |  |  |  |  |  |
| Case | Age(Y) | Sex | LysoGb_3_ (N < 0.6) | LysoHexCer  (N < 3.3) | LysoSM (N < 1.9) | LysoSM509  (N < 7.0) | LysoGM1  (N < 0.07) | LysoGM2 | Comments | *GLA* mutation | |
| 1 | 41.9 | MALE | 77.0 | 0.4 | 0.3 | 0.2 | < 0.07 | Not detected | Classical form | c.692A>G | p.Asp231Gly |
| 2 | 0.3 | MALE | 47.8 | 0.4 | 0.2 | 1.8 | < 0.07 | Not detected | Classical form | c.1000G(-1)>A |  |
| 3 | 48.0 | MALE | 100.8 | 0.6 | 0.3 | 3.5 | < 0.07 | Not detected | Classical form | c.1000G(-1)>A |  |
| 4 | 34.0 | MALE | 57.4 | 0.7 | 0.4 | 2.2 | < 0.07 | Not detected | Classical form | c.840A>C | p.Gln280His |
| 5 | 42.8 | MALE | 81.3 | 0.3 | 0.3 | 4.0 | < 0.07 | Not detected | Classical form | exon 2 del |  |
| 6 | 48.5 | MALE | 67.4 | 1.5 | 0.2 | 1.1 | < 0.07 | Not detected | Classical form | c.802-3_802-2del |  |
| 7 | 16.9 | MALE | 63.2 | 0.6 | 0.2 | 5.3 | < 0.07 | Not detected | Classical form | c.802-3_802-2del |  |
| 8 | 56.1 | MALE | 6.3 | 0.6 | 0.4 | 1.4 | < 0.07 | Not detected | Variant form | c.337T>C | p.Phe113Leu |
| 9 | 34.3 | MALE | 3.3 | 1.0 | 0.3 | 2.4 | < 0.07 | Not detected | Variant form | c.337T>C | p.Phe113Leu |
| 10 | 60.3 | MALE | 3.0 | 0.4 | 0.2 | 1.4 | < 0.07 | Not detected | Variant form | c.593T>C | p.Ile198Thr |
| 11 | 32.4 | MALE | 5.0 | 2.1 | ND | 1.2 | < 0.07 | Not detected | Variant form | c.644A>G | p.Asn215Ser |
| 12 | 24.6 | MALE | 9.4 | 0.4 | 0.4 | 10.1 | < 0.07 | Not detected | Variant form | c.902 G>A | p.Arg301Gln |
| 13 | 29.0 | MALE | 1.8 | 0.8 | 0.4 | 1.1 | < 0.07 | Not detected | Variant form | c.1088G>A | p.Arg363His |
| 14 | 27.1 | FEMALE | 1.2 | 0.6 | 0.2 | 4.3 | < 0.07 | Not detected | Classical form | c.59_73del | p.Ala20_Trp24del |
| 15 | 24.2 | FEMALE | 4.7 | 0.3 | 0.2 | 0.6 | < 0.07 | Not detected | Classical form | c.797A>T | p.Asp266Val |
| 16 | 55.1 | FEMALE | 1.9 | 0.4 | 0.2 | 0.7 | < 0.07 | Not detected | Classical form | c.797A>T | p.Asp266Val |
| 17 | 36.0 | FEMALE | 3.0 | 0.8 | 0.3 | 1.0 | < 0.07 | Not detected | Classical form | c.548G>C | p.Gly183Ala |
| 18 | 56.1 | FEMALE | 1.1 | 0.5 | 0.4 | 4.3 | < 0.07 | Not detected | Classical form | c.548G>C | p.Gly183Ala |
| 19 | 49.4 | FEMALE | 1.4 | 0.5 | 0.3 | 1.4 | < 0.07 | Not detected | Classical form | c.1117G>A | p.Gly373Ser |
| 20 | 49.0 | FEMALE | 3.9 | 1.4 | 0.4 | 1.8 | < 0.07 | Not detected | Classical form | c.137A>G | p.His46Arg |
| 21 | 66.1 | FEMALE | 9.0 | 2.0 | 0.2 | ND | < 0.07 | Not detected | Classical form | c.729G>C | p.Leu243Phe |
| 22 | 26.7 | FEMALE | 3.6 | 1.3 | ND | ND | < 0.07 | Not detected | Classical form | c.899T>C | p.Leu300Pro |
| 23 | 28.1 | FEMALE | 2.3 | 0.7 | ND | 1.9 | < 0.07 | Not detected | Classical form | c.899T>C | p.Leu300Pro |
| 24 | 62.4 | FEMALE | 5.4 | 0.3 | 0.3 | 2.1 | < 0.07 | Not detected | Classical form | c.214delA | p.Met72Trpfs*49 |
| 25 | 33.7 | FEMALE | 4.4 | 0.4 | 0.3 | 3.5 | < 0.07 | Not detected | Classical form | c.214delA | p.Met72Trpfs*49 |
| 26 | 62.1 | FEMALE | 8.5 | 1.8 | 0.4 | 2.2 | < 0.07 | Not detected | Classical form | c.840A>C | p.Gln280His |
| 27 | 63.6 | FEMALE | 6.8 | 0.5 | 0.4 | 1.2 | < 0.07 | Not detected | Classical form | c.679C>T | p.Arg227* |
| 28 | 13.0 | FEMALE | 6.3 | 2.3 | ND | 2.9 | < 0.07 | Not detected | Classical form | c.679C>T | p.Arg227* |
| 29 | 26.3 | FEMALE | 7.9 | 0.6 | 0.3 | 1.7 | < 0.07 | Not detected | Classical form | c.901C>T | p.Arg301* |
| 30 | 49.3 | FEMALE | 3.7 | 0.7 | 0.4 | 3.6 | < 0.07 | Not detected | Classical form | c.901C>T | p.Arg301* |
| 31 | 52.5 | FEMALE | 2.6 | 0.5 | 0.2 | 0.6 | < 0.07 | Not detected | Classical form | c.901C>T | p.Arg301* |
| 32 | 68.0 | FEMALE | 2.8 | 0.3 | 0.3 | 0.9 | < 0.07 | Not detected | Classical form | c.901C>T | p.Arg301* |
| 33 | 51.7 | FEMALE | 1.0 | 0.9 | 0.5 | 2.8 | < 0.07 | Not detected | Classical form | c.1087C>T | p.Arg363Cys |
| 34 | 27.0 | FEMALE | 8.3 | 0.4 | 1.3 | 2.9 | < 0.07 | Not detected | Classical form | c.786G>A | p.Trp262* |
| 35 | 29.8 | FEMALE | 3.0 | 0.5 | 0.2 | 1.9 | < 0.07 | Not detected | Classical form | exon 2 del |  |
| 36 | 54.0 | FEMALE | 8.5 | 0.3 | 0.2 | 2.5 | < 0.07 | Not detected | Classical form | c.802-3_802-2del |  |
| 37 | 18.8 | FEMALE | 5.4 | 0.4 | 0.3 | 1.1 | < 0.07 | Not detected | Classical form | c.802-3_802-2del |  |
| 38 | 51.0 | FEMALE | 31.2 | 0.5 | 0.2 | 1.4 | < 0.07 | Not detected | Classical form | c.802-3_802-2del |  |
| 39 | 34.6 | FEMALE | 6.9 | 0.4 | 0.3 | 1.2 | < 0.07 | Not detected | Classical form | IVS1 +1 G>A | c.194+1G>A |
| 40 | 33.7 | FEMALE | 0.6 | 0.4 | 0.4 | 2.8 | < 0.07 | Not detected | Variant form | c.337T>C | p.Phe113Leu |
| 41 | 60.7 | FEMALE | 2.8 | 1.0 | 0.5 | 2.2 | < 0.07 | Not detected | Variant form | c.337T>C | p.Phe113Leu |
| 42 | 51.8 | FEMALE | 1.6 | 0.6 | 0.4 | 2.0 | < 0.07 | Not detected | Variant form | c.644A>G | p.Asn215Ser |
| **Gaucher disease** | |  |  |  |  |  |  |  |  |  |  |
| 43 | 1.2 | MALE | 2.3 | 115.0 | 0.3 | 14.3 | < 0.07 | Not detected | Type 1 |  |  |
| 44 | 1.9 | MALE | 0.9 | 45.7 | 0.6 | 19.4 | < 0.07 | Not detected | Type 1 |  |  |
| 45 | 3.6 | MALE | 2.1 | 224.0 | 1.0 | 8.6 | < 0.07 | Not detected | Type 1 |  |  |
| 46 | 6.5 | FEMALE | 2.4 | 275.0 | 2.3 | 3.8 | < 0.07 | Not detected | Type 1 |  |  |
| 47 | 11.7 | MALE | 1.4 | 249.0 | 1.4 | 38.7 | < 0.07 | Not detected | Type 1 |  |  |
| 48 | 30.5 | FEMALE | 2.1 | 427.0 | 2.3 | 3.0 | < 0.07 | Not detected | Type 1 |  |  |
| 49 | 37.2 | FEMALE | 2.1 | 302.0 | 1.7 | 9.6 | < 0.07 | Not detected | Type 1 |  |  |
| 50 | 50.2 | MALE | 2.6 | 154.0 | 1.1 | 1.5 | < 0.07 | Not detected | Type 1 |  |  |
| **Saposine C deficiency** | | |  |  |  |  |  |  |  |  |  |
| 51 | 27.1 | FEMALE | 1.9 | 75.8 | 1.5 | 1.2 | < 0.07 | Not detected |  |  |  |
| **Krabbe disease** | | |  |  |  |  |  |  |  |  |  |
| 52 | 0.4 | MALE | 0.3 | 21.6 | 0.2 | 0.6 | < 0.07 | Not detected |  |  |  |
| 53 | 0.4 | MALE | 0.1 | 11.5 | 0.1 | 0.4 | < 0.07 | Not detected |  |  |  |
| 54 | 0.5 | MALE | 0.2 | 14.0 | 0.5 | 1.4 | < 0.07 | Not detected |  |  |  |
| 55 | 0.7 | FEMALE | 0.2 | 9.7 | 0.4 | 1.3 | < 0.07 | Not detected |  |  |  |
| 56 | 0.6 | MALE | 0.1 | 18.5 | 0.3 | 0.9 | < 0.07 | Not detected |  |  |  |
| 57 | 1.0 | FEMALE | 0.4 | 10.8 | 0.2 | 0.1 | < 0.07 | Not detected |  |  |  |
| 58 | 1.0 | FEMALE | 0.1 | 9.3 | 0.1 | 1.2 | < 0.07 | Not detected |  |  |  |
| **Niemann-Pick type A/B disease** | | | |  |  |  |  |  |  |  |  |
| 59 | 0.7 | FEMALE | 0.1 | 0.5 | 11.3 | 166.1 | < 0.07 | Not detected | Type A |  |  |
| 60 | 0.8 | MALE | 0.1 | 1.0 | 69.6 | 182.0 | < 0.07 | Not detected | Type A |  |  |
| 61 | 1.0 | FEMALE | 0.3 | 3.3 | 41.8 | 344.4 | < 0.07 | Not detected | Type B |  |  |
| 62 | 1.6 | MALE | 0.2 | 1.7 | 23.1 | 327.6 | < 0.07 | Not detected | Type B |  |  |
| 63 | 13.2 | MALE | 0.5 | 2.3 | 20.6 | 126.8 | < 0.07 | Not detected | Type B |  |  |
| 64 | 14.2 | MALE | 0.3 | 0.4 | 16.1 | 127.8 | < 0.07 | Not detected | Type B |  |  |
| 65 | 17.1 | MALE | 0.0 | 0.8 | 17.3 | 141.0 | < 0.07 | Not detected | Type B |  |  |
| 66 | 19.9 | MALE | 0.3 | 0.5 | 15.8 | 168.0 | < 0.07 | Not detected | Type B |  |  |
| 67 | 29.3 | MALE | 0.3 | 1.0 | 9.6 | 214.6 | < 0.07 | Not detected | Type B |  |  |
| 68 | 32.8 | FEMALE | 0.1 | 0.5 | 8.0 | 236.7 | < 0.07 | Not detected | Type B |  |  |
| 69 | 41.0 | MALE | 0.0 | 0.6 | 10.7 | 363.8 | < 0.07 | Not detected | Type B |  |  |
| 70 | 48.8 | FEMALE | 0.3 | 0.4 | 9.2 | 233.8 | < 0.07 | Not detected | Type B |  |  |
| 71 | 54.1 | MALE | 0.2 | 0.5 | 13.0 | 242.1 | < 0.07 | Not detected | Type B |  |  |
| 72 | 73.1 | FEMALE | 0.1 | 0.5 | 12.0 | 227.9 | < 0.07 | Not detected | Type B |  |  |
| **Niemann-Pick type C disease** | | | |  |  |  |  |  |  |  |  |
| 73 | 37.7 | MALE | 0.6 | 1.4 | 2.1 | 138.1 | < 0.07 | Not detected | *NPC1* |  |  |
| 74 | 10.3 | FEMALE | 0.2 | 0.9 | 0.6 | 101.5 | < 0.07 | Not detected | *NPC1* |  |  |
| 75 | 8.3 | MALE | 0.8 | 0.2 | 0.7 | 141.7 | < 0.07 | Not detected | *NPC1* |  |  |
| 76 | 2.9 | FEMALE | 0.1 | 0.6 | 1.4 | 348.6 | < 0.07 | Not detected | *NPC1* |  |  |
| 77 | 1.6 | FEMALE | 0.4 | 0.4 | 0.3 | 129.2 | < 0.07 | Not detected | *NPC1* |  |  |
| 78 | 1.0 | MALE | 0.2 | 1.2 | 0.6 | 194.8 | < 0.07 | Not detected | *NPC1* |  |  |
| 79 | 0.7 | MALE | 0.0 | 1.1 | 0.5 | 118.8 | < 0.07 | Not detected | *NPC1* |  |  |
| 80 | 0.2 | MALE | 0.2 | 2.4 | 0.8 | 204.1 | < 0.07 | Not detected | *NPC1* |  |  |
| **GM1 Gangliosidosis** | | |  |  |  |  |  |  |  |  |  |
| 81 | 0.1 | MALE | 0.4 | 2.6 | 0.8 | 1.1 | 8.6 | Not detected |  |  |  |
| 82 | 0.3 | MALE | 0.1 | 1.6 | 0.5 | 1.9 | 1.5 | Not detected |  |  |  |
| 83 | 0.5 | FEMALE | 0.2 | 3.7 | 1.2 | 1.9 | 39.9 | Not detected |  |  |  |
| 84 | 1.8 | MALE | 0.1 | 2.2 | 0.6 | 1.4 | 33.9 | Not detected |  |  |  |
| 85 | 1.8 | MALE | 0.8 | 2.5 | 0.5 | 4.7 | 2.2 | Not detected |  |  |  |
| 86 | 26.2 | FEMALE | 0.7 | 0.7 | 0.4 | 0.3 | < 0.07 | Not detected |  |  |  |
| **GM2 Gangliosidosis** | | |  |  |  |  |  |  |  |  |  |
| 87 | 0.6 | FEMALE | 0.9 | 0.8 | 0.1 | 3.0 | < 0.07 | 6.4 | Sandhoff disease |  |  |
| 88 | 0.8 | MALE | 0.8 | 0.4 | 0.2 | 0.5 | < 0.07 | 9.9 | Sandhoff disease |  |  |
| 89 | 1.4 | MALE | 0.1 | 0.2 | 0.3 | 1.6 | < 0.07 | 118 | Sandhoff disease |  |  |
| 90 | 1.4 | FEMALE | 0.9 | 0.6 | 0.4 | 1.0 | < 0.07 | 3.4 | Sandhoff disease |  |  |
| 91 | 1.6 | MALE | 3.0 | 0.5 | 0.2 | 0.0 | < 0.07 | 4.6 | Sandhoff disease |  |  |
| 92 | 2.6 | MALE | 0.2 | 1.2 | 0.3 | 1.2 | < 0.07 | Not detected | Sandhoff disease |  |  |
| 93 | 11.5 | MALE | 0.9 | 0.8 | 0.3 | 0.8 | < 0.07 | 24.2 | Sandhoff disease |  |  |
| 94 | 25.2 | FEMALE | 0.7 | 1.2 | 0.4 | 2.1 | < 0.07 | Not detected | Sandhoff disease |  |  |
| 95 | 44.0 | FEMALE | 0.4 | 0.1 | 0.2 | 1.7 | < 0.07 | 0.7 | Sandhoff disease |  |  |
| 96 | 0.0 | MALE | 0.2 | 0.9 | 0.4 | 1.2 | < 0.07 | 14.5 | Tay-Sachs disease |  |  |
| 97 | 4.3 | MALE | 0.6 | 0.8 | 0.3 | 1.4 | < 0.07 | 0.6 | Tay-Sachs disease |  |  |
| 98 | 5.1 | MALE | 0.2 | 1.4 | 0.4 | 1.8 | < 0.07 | 0.3 | Tay-Sachs disease |  |  |
| 99 | 40.0 | MALE | 0.8 | 1.6 | 0.6 | 0.6 | < 0.07 | Not detected | Tay-Sachs disease |  |  |
| **OTHER INBORN ERROR OF METABOLISM** | | | | |  |  |  |  |  |  |  |
| **Lysosomal acid lipase deficiency** | | | |  |  |  |  |  |  |  |  |
| 100 | 19.3 | MALE | 0.2 | 0.9 | 0.4 | 9.9 | < 0.07 | Not detected | Lysosomal acid lipase deficiency |  |  |
| 101 | 17.1 | MALE | 0.3 | 0.9 | 0.8 | 10.3 | < 0.07 | Not detected | Lysosomal acid lipase deficiency |  |  |
| 102 | 0.2 | FEMALE | 0.5 | 1.4 | 0.4 | 11.6 | < 0.07 | Not detected | Lysosomal acid lipase deficiency |  |  |
| **Other lysosomal storage diseases** | | | |  |  |  |  |  |  |  |  |
| 103 | 5.6 | MALE | 0.2 | 1.5 | 0.4 | 1.0 | < 0.07 | Not detected | Metachromatic leukodystrophy |  |  |
| 104 | 5.8 | MALE | 0.3 | 0.8 | 0.4 | 0.7 | < 0.07 | Not detected | Metachromatic leukodystrophy |  |  |
| 105 | 1.0 | MALE | 3.1 | 1.0 | 0.5 | 1.7 | < 0.07 | Not detected | Mucoplysaccharidosis type I |  |  |
| 106 | 0.1 | MALE | 0.3 | 0.8 | 0.6 | 1.5 | < 0.07 | Not detected | Mucoplysaccharidosis type II |  |  |
| 107 | 0.1 | MALE | 0.8 | 1.2 | 0.5 | 1.1 | < 0.07 | Not detected | Pompe disease |  |  |
| 108 | 69.0 | MALE | 0.4 | 1.3 | 0.7 | 2.3 | < 0.07 | Not detected | Pompe disease |  |  |
| 109 | 62.7 | MALE | 0.6 | 0.9 | 0.8 | 1.6 | < 0.07 | Not detected | Pompe disease |  |  |
| 110 | 49.8 | FEMALE | 0.4 | 1.7 | 0.8 | 1.7 | < 0.07 | Not detected | Pompe disease |  |  |
| 111 | 7.6 | MALE | 0.4 | 0.8 | 0.4 | 1.2 | < 0.07 | Not detected | Pompe disease |  |  |
| 112 | 15.2 | MALE | 0.2 | 0.5 | 0.4 | 1.0 | < 0.07 | Not detected | Mucolipidosis type III |  |  |
| **Peroxisomal disorders** | | | |  |  |  |  |  |  |  |  |
| 113 | 51.7 | MALE | 0.5 | 0.5 | 0.9 | 1.6 | < 0.07 | Not detected | X-linked adrenoleukodystrophy |  |  |
| 114 | 6.4 | MALE | 1.3 | 2.1 | 6.5 | 0.1 | < 0.07 | Not detected | X-linked adrenoleukodystrophy |  |  |
| 115 | 7.4 | MALE | 0.2 | 0.5 | 0.7 | 8.3 | < 0.07 | Not detected | X-linked adrenoleukodystrophy |  |  |
| 116 | 0.1 | MALE | 0.1 | 0.6 | 0.2 | 3.0 | < 0.07 | Not detected | Peroxisomal biogenesis defects |  |  |
| 117 | 16.5 | MALE | 0.4 | 3.5 | 0.6 | 2.2 | < 0.07 | Not detected | Peroxisomal biogenesis defects |  |  |
| 118 | 1.3 | MALE | 0.3 | 1.1 | 0.3 | 4.6 | < 0.07 | Not detected | Peroxisomal biogenesis defects |  |  |
| 119 | 15.9 | FEMALE | 0.1 | 0.4 | 0.3 | 1.6 | < 0.07 | Not detected | Peroxisomal biogenesis defects |  |  |
| 120 | 87.2 | FEMALE | 0.4 | 1.4 | 2.0 | 0.5 | < 0.07 | Not detected | Adrenomyeloneuropathy |  |  |
| 121 | 49.0 | FEMALE | 0.1 | 2.3 | 0.7 | 1.2 | < 0.07 | Not detected | Adrenomyeloneuropathy |  |  |
| 122 | 69.4 | FEMALE | 0.1 | 0.9 | 2.1 | 0.4 | < 0.07 | Not detected | Adrenomyeloneuropathy |  |  |
