# Supplementary material for: LC-MS/MS multiplex analysis of lysosphingolipids in plasma and amniotic fluid: A novel tool for the screening of sphingolipidoses and Niemann-Pick type C disease
Source: PLoS One. 2017 Jul 27;12(7):e0181700. doi: 10.1371/journal.pone.0181700 (PMC5531455; doi:10.1371/journal.pone.0181700)
Supplement: S2 Table — Recovery rates within the range of 85–115%. and RSD below 20% were considered acceptable. (DOCX) [file pone.0181700.s005.docx]

|  | Low added concentration | | | Medium added concentration | | | High added concentration | | |
| --- | --- | --- | --- | --- | --- | --- | --- | --- | --- |
|  | Concentration (nmol/L) | Recovery (%) | RSD  (%) | Concentration (nmol/L) | Recovery  (%) | RSD  (%) | Concentration (nmol/L) | Recovery (%) | RSD (%) |
| LysoGb_3_ | 39.6 | 96 | 9.1 | 79.2 | 97 | 18.9 | 396 | 101 | 4.6 |
| LysoGlcCer | 21.5 | 86 | 5.9 | 43.0 | 85 | 19.8 | 215 | 88 | 6.9 |
| LysoSM d18:1 | 2.3 | 87 | 15.4 | 4.7 | 86 | 19.1 | 23.3 | 88 | 15.8 |
| LysoGM1 | 7.8 | 53 | 57.0 | 15.6 | 48 | 61.5 | 78.1 | 46 | 39.9 |
