# Supplementary material for: LC-MS/MS multiplex analysis of lysosphingolipids in plasma and amniotic fluid: A novel tool for the screening of sphingolipidoses and Niemann-Pick type C disease
Source: PLoS One. 2017 Jul 27;12(7):e0181700. doi: 10.1371/journal.pone.0181700 (PMC5531455; doi:10.1371/journal.pone.0181700)
Supplement: S3 Table — Biases below 20% were considered acceptable. (DOCX) [file pone.0181700.s006.docx]

|  | LysoGb_3_ | LysoHexCer | LysoSM |
| --- | --- | --- | --- |
| EDTA | 0.8 | 0.6 | 0.4 |
| Dry tube | 0.9 | 0.9 | 0.4 |
| Bias % | 10% | 58% | 0% |
| Heparin | 0.8 | 0.5 | 0.3 |
| Bias % | 8% | 10% | 1% |
