# Supplementary material for: LC-MS/MS multiplex analysis of lysosphingolipids in plasma and amniotic fluid: A novel tool for the screening of sphingolipidoses and Niemann-Pick type C disease
Source: PLoS One. 2017 Jul 27;12(7):e0181700. doi: 10.1371/journal.pone.0181700 (PMC5531455; doi:10.1371/journal.pone.0181700)
Supplement: S4 Table — Biases below 20% were considered acceptable. (DOCX) [file pone.0181700.s007.docx]

|  | EDTA |  |  | Dry tube |  |  | Heparine |  |  |
| --- | --- | --- | --- | --- | --- | --- | --- | --- | --- |
| Plasma/serum at room temperature | LysoGb_3_ | LysoHexCer | LysoSM | LysoGb_3_ | LysoHexCer | LysoSM | LysoGb_3_ | LysoHexCer | LysoSM |
| J0 | 0.8 | 0.6 | 0.4 | 0.9 | 0.9 | 0.4 | 0.8 | 0.6 | 0.4 |
| J1 | 0.9 | 0.6 | 0.4 | 0.8 | 0.9 | 0.4 | 0.8 | 0.6 | 0.4 |
| *Bias %* | *12.5* | *0.0* | *0.0* | *-11.1* | *0.0* | *0.0* | *0.0* | *0.0* | *0.0* |
| J2 | 0.8 | 0.7 | 0.4 | 0.9 | 0.8 | 0.4 | 0.8 | 0.6 | 0.4 |
| *Bias %* | *0.0* | *16.6* | *0.0* | *0.0* | *-11.1* | *0.0* | *0.0* | *0.0* | *0.0* |
| Refrigerated plasma/serum | LysoGb_3_ | LysoHexCer | LysoSM | LysoGb_3_ | LysoHexCer | LysoSM | LysoGb_3_ | LysoHexCer | LysoSM |
| J0 | 0.8 | 0.6 | 0.4 | 0.9 | 0.9 | 0.4 | 0.8 | 0.6 | 0.4 |
| J1 | 0.7 | 0.6 | 0.4 | 0.9 | 0.8 | 0.4 | 0.9 | 0.6 | 0.4 |
| *Bias %* | *-12.5* | *0.0* | *0.0* | *0.0* | *-11.1* | *0.0* | *12.5* | *0.0* | *0.0* |
| J2 | 0.8 | 0.6 | 0.4 | 0.9 | 1.0 | 0.4 | 0.8 | 0.6 | 0.4 |
| *Bias %* | *0.0* | *0.0* | *0.0* | *0.0* | *11.1* | *0.0* | *0.0* | *0.0* | *0.0* |
| Total blood | LysoGb_3_ | LysoHexCer | LysoSM | LysoGb_3_ | LysoHexCer | LysoSM | LysoGb_3_ | LysoHexCer | LysoSM |
| J0 | 0.6 | 0.3 | 0.3 | 0.6 | 0.7 | 0.3 | 0.6 | 0.5 | 0.3 |
| J1 | 0.6 | 0.3 | 0.3 | 0.6 | 0.8 | 0.3 | 0.7 | 0.6 | 0.3 |
| *Bias %* | *0.0* | *0.0* | *0.0* | *0.0* | *14.3* | *0.0* | *16.6* | *20.0* | *0.0* |
| J2 | 0.6 | 1.2 | 0.3 | 0.5 | 1.6 | 0.3 | 0.6 | 0.6 | 0.3 |
| *Bias %* | *0.0* | *300.0* | *0.0* | *-16.6* | *128.6* | *0.0* | *0.0* | *20.0* | *0.0* |
| J3 | 0.6 | 1.7 | 0.4 | / | / | / | 0.6 | 0.7 | 0.3 |
| *Bias %* | *0.0* | *466.0* | *33.3* | */* | */* | */* | *0.0* | *40.0* | *0.0* |
| J7 | 0.6 | 2.1 | 0.5 | / | / | / | 0.4 | 1.5 | 0.4 |
| *Bias %* | *0.0* | *600.0* | *66.6* | */* | */* | */* | *-33.3* | *200.0* | *33.3* |
