# Supplementary material for: LC-MS/MS multiplex analysis of lysosphingolipids in plasma and amniotic fluid: A novel tool for the screening of sphingolipidoses and Niemann-Pick type C disease
Source: PLoS One. 2017 Jul 27;12(7):e0181700. doi: 10.1371/journal.pone.0181700 (PMC5531455; doi:10.1371/journal.pone.0181700)
Supplement: S5 Table — (DOCX) [file pone.0181700.s008.docx]

| Hemoglobin concentration | LysoGb_3_ | LysoHexCer | LysoSM | LysoSM509 |
| --- | --- | --- | --- | --- |
| 0 g/L | 0.2 | 0.5 | 0.6 | 3.3 |
| 2.4 g/L | 0.1 | 0.6 | 0.7 | 3.3 |
| 3.0 g/L | 0.1 | 0.6 | 0.9 | 3.7 |
| 4.7 g/L | 0.1 | 0.7 | 0.9 | 3.8 |
| 7.9 g/L | 0.4 | 0.6 | 0.8 | 3.4 |
