# Supplementary material for: LC-MS/MS multiplex analysis of lysosphingolipids in plasma and amniotic fluid: A novel tool for the screening of sphingolipidoses and Niemann-Pick type C disease
Source: PLoS One. 2017 Jul 27;12(7):e0181700. doi: 10.1371/journal.pone.0181700 (PMC5531455; doi:10.1371/journal.pone.0181700)
Supplement: S6 Table — NS: non-significant. (DOCX) [file pone.0181700.s009.docx]

|  | Sex | N | Mean | sd | *p* |
| --- | --- | --- | --- | --- | --- |
| LysoGb_3_ | male | 54 | 0.2 | 0.18 | NS |
|  | female | 88 | 0.2 | 0.17 |  |
| LysoHexCer | male | 54 | 0.4 | 0.23 | NS |
|  | female | 88 | 0.5 | 0.36 |  |
| LysoSM | male | 54 | 0.2 | 0.12 | NS |
|  | female | 88 | 0.2 | 0.09 |  |
| LysoSM509 | male | 54 | 1.4 | 1.1 | NS |
|  | female | 88 | 1.5 | 1.0 |  |
