# Supplementary material for: LC-MS/MS multiplex analysis of lysosphingolipids in plasma and amniotic fluid: A novel tool for the screening of sphingolipidoses and Niemann-Pick type C disease
Source: PLoS One. 2017 Jul 27;12(7):e0181700. doi: 10.1371/journal.pone.0181700 (PMC5531455; doi:10.1371/journal.pone.0181700)
Supplement: S7 Table — NS: non-significant. y: year. (DOCX) [file pone.0181700.s010.docx]

|  | Age | N | mean | sd | *p* |
| --- | --- | --- | --- | --- | --- |
| LysoGb_3_ | < 10 y | 24 | 0.1 | 0.18 | *p* < 0.01 |
|  | > 10 y | 118 | 0.2 | 0.17 |  |
| LysoHexCer | < 10 y | 24 | 0.7 | 0.49 | NS |
|  | > 10 y | 118 | 0.7 | 0.53 |  |
| LysoSM | < 10 y | 24 | 0.3 | 0.23 | *p* < 0.05 |
|  | > 10 y | 118 | 0.3 | 0.25 |  |
| LysoSM509 | < 10 y | 24 | 1.3 | 1.44 | NS |
|  | > 10 y | 118 | 1.5 | 0.95 |  |
